# Effects of Inspired Oxygen Concentrations During Cardiopulmonary Bypass on the Pulmonary Function of Patients Undergoing a Modified Morrow Procedure via a Small Right Axillary Incision

**DOI:** 10.1155/anrp/9078621

**Published:** 2026-06-30

**Authors:** Linting Xu, Lixin Wang, Meijuan Yan, Hanwei Wei, Tian Jiang, Haozhou Wang, Jinchen Guo, Yong Cui

**Affiliations:** ^1^ Center for Rehabilitation Medicine, Department of Anesthesiology, Zhejiang Provincial People’s Hospital, Affiliated People’s Hospital, Hangzhou Medical College, Hangzhou, 310000, Zhejiang, China, hznu.edu.cn; ^2^ Department of Anesthesiology, The First Affiliated Hospital of Shantou University Medical College, Shantou, 515000, Guangdong, China, stuh.com.cn; ^3^ Heart Center, Department of Cardiovascular Surgery, Zhejiang Provincial People’s Hospital (Affiliated People’s Hospital), Hangzhou Medical College, Hangzhou, 310000, Zhejiang, China, hznu.edu.cn

**Keywords:** hypertrophic obstructive cardiomyopathy, modified Morrow procedure, pulmonary function, right axillary small incision cardiac surgery, ultra-fast-track cardiac anesthesia

## Abstract

**Background:**

Patients with hypertrophic obstructive cardiomyopathy (HOCM) undergoing minimally invasive modified Morrow procedure may be vulnerable to postoperative pulmonary dysfunction. During cardiopulmonary bypass (CPB), lung‐protective ventilation has been proposed, but the optimal inspired oxygen concentration under this strategy remains unclear. This study aimed to compare different inspired oxygen concentrations under a fixed lung‐protective ventilation strategy during CPB.

**Methods:**

This was a prospective clinical observation study involving 97 patients scheduled for modified Morrow procedures via a small right axillary incision between January 2023 and November 2023 at a single center. Patients were randomized to receive CPB ventilation with FiO_2_ of 30%, 50%, or 100% under a standardized ultra‐low‐tidal‐volumelung‐protective strategy. The primary outcomes included the Horowitz Index (HI = PaO_2_/FiO_2_), respiratory index (RI = P(A‐a)O_2_/PaO_2_), and alveolar–arterial oxygen pressure difference [P(A‐a)O_2_] at six different time points within the first 24 h postoperatively, as well as postoperative pulmonary complications. Secondary outcomes included plasma levels of inflammatory cytokines, endothelial glycocalyx (eGC) components, and measures of postoperative rehabilitation and other complications.

**Results:**

There were no significant differences in terms of postoperative complications and rehabilitation among the three groups with different inspired oxygen concentrations under a fixed lung‐protective ventilation strategy during CPB. Compared with the 100% FiO_2_ group, the 30% FiO_2_ group exhibited higher HI at 6 h and 24 h postoperatively with lower P(A‐a)O_2_ at 18 h and 24 h postoperatively and a lower RI at 24 h postoperatively. In addition, the 30% FiO_2_ group showed lower plasma levels of inflammatory cytokines and eGC.

**Conclusion:**

These findings indicate that the use of 30% 100% FiO_2_ group with ultra‐low tidal volume ventilation during CPB in patients undergoing a modified Morrow procedure via a small right axillary incision was safe and associated with improved early postoperative oxygenation and attenuated inflammatory response.

**Trial Registration:** Chinese Clinical Trial Registry: ChiCTR2400084443

## 1. Introduction

For hypertrophic obstructive cardiomyopathy (HOCM), surgical septal myectomy is regarded as the gold standard treatment [1]. Since 2018, our team has performed over 200 cases annually of minimally invasive electrosurgical septal myectomy (MESM)—a modified Morrow procedure via a right axillary mini‐incision under cardiopulmonary bypass (CPB) [2].

In HOCM patients, left ventricular outflow tract obstruction elevates left ventricular end‐diastolic pressure, retrogradely increasing left atrial and pulmonary venous pressures and causing pulmonary congestion. Consequently, these patients often have compromised baseline pulmonary function. CPB induces ischemia–reperfusion injury and systemic inflammation, disrupting the alveolar–capillary barrier [3]. Both HOCM pathophysiology and CPB‐related insults impair postoperative pulmonary recovery, underscoring the importance of perioperative lung‐protective strategies during CPB [4].

Lung‐protective ventilation—typically involving low tidal volume (VT), positive end‐expiratory pressure (PEEP), and recruitment maneuvers—has become the cornerstone of intraoperative respiratory management [5]. However, the optimal inspired oxygen fraction (FiO_2_) during CPB remains controversial. Although high FiO_2_ is traditionally used to prevent hypoxemia, evidence suggests it may delay pulmonary recovery and exacerbate inflammation [6], though some studies report no significant differences compared with low FiO_2_ [7]. Notably, most existing studies have focused on conventional median sternotomy, leaving the optimal FiO_2_ during CPB uncertain in the minimally invasive modified Morrow procedure via a small right axillary incision.

To address this gap, we designed a prospective randomized controlled trial investigating the effects of different FiO_2_ levels (30%, 50%, and 100%) administered during CPB on postoperative pulmonary function in HOCM patients undergoing MESM via a right axillary incision. All patients received a uniform ventilation strategy involving ultra‐low VT, low‐frequency ventilation, and PEEP.

## 2. Methods

### 2.1. Study Design and Participants

This study is a prospective, double‐blind, randomized controlled trial approved by the hospital’s ethics committee (2022KY062). Between January 2023 and November 2023, 97 patients who were scheduled for right axillary incision‐modified Morrow procedure at Zhejiang Provincial People’s Hospital were enrolled. Ultra‐fast‐track cardiac anesthesia (UFTCA) is defined as a perioperative management strategy characterized by extubation within 1 h postoperatively, optimized anesthetic protocols to facilitate rapid recovery, and multimodal analgesia to minimize opioid use. UFTCA was implemented. Informed consent was obtained from all patients and their families. The inclusion criteria were as follows: (1) physical status Grade III or below according to the criteria of the American Society of Anesthesiologists (ASA); (2) cardiac functional grade III or below according to criteria of the New York Heart Association (NYHA); (3) age of 18–65 years; (4) left ventricular diastolic function grade II or below; and (5) no contraindications for nerve blockade. The exclusion criteria were as follows: (1) patients refused to participate; (2) severe communication barriers; (3) severe coagulation abnormalities; (4) contraindications for nerve blockade; (5) allergy to dexmedetomidine and ropivacaine; and (6) severe subgroup 1 of pulmonary hypertension (PH): mean pulmonary artery pressure > 55 mmHg. In EuroSCORE II, PH is defined as systolic pulmonary artery pressure > 55 mmHg as a binary variable, and exceeding this threshold significantly increases the predicted risk of operative mortality [8]; (7) NYHA Class IV. The following exclusion criteria were applied during the study: (1) CPB time (duration of CPB > 200 min; (2) aortic cross‐clamping time > 156 min; (3) unsuccessful extubation within 1 h postoperatively; (4) reoperation; and (5) intraoperative second‐run CPB.

### 2.2. Randomization and Blinding

This was a prospective, randomized, patient‐, and assessor‐blinded trial. The sample size was calculated using G ∗ Power software (Version 3.1.9.7) based on the means and standard deviations of the primary outcome (Horowitz Index [HI]). Assuming a two‐sided *α* of 0.05, a power (1‐β) of 80%, and a dropout rate of 10%, the calculated total sample size was 90 patients, requiring a minimum of 30 patients per group. A total of 97 eligible patients were sequentially numbered 1 to 97 and randomly allocated to three groups in a 1:1:1 ratio using a computer‐generated random sequence (SPSS 26.0) prepared by an independent statistician. Allocation was concealed using sequentially numbered, opaque, sealed envelopes. Patients were unaware of their group assignment throughout the study. Outcome assessors were blinded to group allocation and had no access to surgical or anesthetic records. All laboratory analyses were performed by technicians blinded to group assignment using coded samples. The three groups received the following interventions during CPB:

Group A (*n* = 32): 30% inspired oxygen concentration, ultra‐low VT lung‐protective ventilation during CPB [VT: 1.5 mL/kg, respiratory rate (RR): 8 breaths/min, PEEP: 5 cmH_2_O, fresh gas flow: 2 L/min]; Group B (*n* = 32): 50% inspired oxygen concentration, ultra‐low VT lung‐protective ventilation during CPB (VT: 1.5 mL/kg, RR: 8 breaths/min, PEEP: 5 cmH_2_O, fresh gas flow: 2 L/min); Group C (*n* = 33): 100% inspired oxygen concentration, ultra‐low VT lung‐protective ventilation during CPB (VT: 1.5 mL/kg, RR: 8 breaths/min, PEEP: 5 cmH_2_O, fresh gas flow: 2 L/min). Anesthesiologists adjusted the oxygen concentrations according to group assignments. The operating room staff (surgeons, perfusionists, and nursing staff) and participating patients, with the exception of the anesthesiologists, were blinded to group allocation. Postoperative follow‐up and data collection were conducted by two cardiac anesthesia nurses who were not involved in intraoperative anesthesia management and remained blinded to group assignments throughout the study.

### 2.3. Anesthesia Protocol

Upon entry into the operating room, all patients underwent routine monitoring of vital signs, including noninvasive blood pressure, electrocardiography (ECG), pulse oxygen saturation (SpO_2_), and anesthesia depth via the entropy index (EI), along with invasive arterial pressure monitoring.

Anesthesia Induction: Anesthesia induction was performed by injection of etomidate emulsion 0.15 mg/kg, propofol injection 1 mg/kg, sufentanil citrate injection 0.2–0.5 μg/kg, and cisatracurium besylate injection 0.3–0.5 mg/kg. The combination of etomidate and propofol was applied to balance hemodynamic stability: Etomidate causes minimal cardiovascular depression, which is suitable for HOCM patients sensitive to hemodynamic fluctuations, while low‐dose propofol reduces the incidence of myoclonus and adrenal suppression caused by etomidate alone. Oral intubation was performed with the help of direct laryngoscopy using a suctionable tracheal tube (an endotracheal tube equipped with a suction channel for removing secretions accumulated above the tracheal tube cuff). Correct tube placement was confirmed by auscultation, followed by fixation and initiation of mechanical ventilation. The parameters of mechanical ventilation were FiO_2_: 50%–80%, VT: 6–8 mL/kg, RR: 10–12 breaths/min, and I:E ratio: 1:2, maintaining end‐tidal carbon dioxide pressure (PetCO_2_) at 35–45 mmHg. Right internal jugular vein cannulation was performed after anesthesia induction, and temperature probes were placed in the nasopharynx and rectum, along with transesophageal echocardiography (TEE) probe insertion.

Right chest wall fascial plane block was performed after anesthesia induction using 0.375% ropivacaine combined with 1 μg/kg dexmedetomidine, under strict aseptic conditions by experienced anesthesiologists.

Anesthesia Maintenance: Anesthesia was maintained by continuous infusion of propofol 150–250 μg/kg·min and remifentanil 0.1–1 μg/kg·min. The anesthesia depth EI was maintained at 40–60. Based on hemodynamic and respiratory parameters and surgical requirements, additional sufentanil of 0.1–0.2 μg/kg was administered intermittently, with a total dose controlled within 1.0–1.5 μg/kg. Cisatracurium besylate was supplemented during aortic clamping and unclamping at doses of 0.1–0.2 mg/kg.

Anesthesia Recovery and Extubation: Patients deemed suitable for UFTCA based on their condition and surgical outcomes discontinued their maintenance medication in a timely fashion. Lung recruitment maneuvers were performed in all patients after weaning from CPB and before tracheal extubation, consisting of 30 cmH_2_O CPAP for 15 s repeated 3 times to reverse atelectasis. Tracheal extubation was then accomplished in awake patients. The extubation criteria included the following: (1) stable hemodynamics without or with minimal vasoactive drug assistance (e.g., norepinephrine ≤ 0.05 μg/kg·min, dobutamine ≤ 5.0 μg/kg·min), absence of refractory arrhythmias (defined as sustained ventricular arrhythmias, or supraventricular arrhythmias with rapid ventricular response that cannot be reversed by antiarrhythmic drugs or electrical cardioversion within 30 min); (2) arterial blood gas analysis showing pH 7.35–7.45 without electrolyte disturbances; (3) absence of significant active bleeding (hemoglobin ≥ 80 g/L, activated clotting time (ACT) restored to preoperative baseline); (4) clear consciousness, able to follow commands (open eyes, extend tongue, grip fist, lift head), and if necessary, residual effects of neuromuscular blockade were antagonized using 1 mg neostigmine and 0.2 mg atropine sulfate injection; and (5) smooth spontaneous breathing, restored airway reflexes, with SPO_2_ ≥ 95%, PaO_2_ ≥ 60 mmHg, and PaCO_2_ 35–45 mmHg upon 100% FiO_2_ prior to extubation.

Postextubation Oxygen Therapy: After extubation, if patients treated with nasal cannula had SPO_2_ values persistently below 95%, PaO_2_ below 80 mmHg, or HI below 200 mmHg, high‐flow nasal cannula (HFNC) was given. Common parameters for HFNC included FiO_2_ 50% and flow 50 L/min, adjusted based on oxygenation status, and discontinued upon improvement.

Postoperative Analgesia: Following tracheal extubation, all patients received intravenous patient‐controlled analgesia. The analgesic solution consisted of sufentanil 200 μg, esketamine 50 mg, droperidol 1 mg, and palonosetron 0.25 mg, diluted with normal saline to a total volume of 200 mL, and was programmed with a lockout interval of 15 min, a single bolus dose of 4 μg of sufentanil, a background infusion rate of 3 μg/h, and a maximum hourly limit of 20 μg. Postoperative pain intensity was evaluated using the visual analog score (VAS). A rescue intravenous bolus of tramadol 50 mg was administered if the VAS score was ≥ 3 after patient‐controlled analgesia.

### 2.4. Outcome Measures

The primary outcome measures included the following: (1) arterial blood gas analysis for HI, RI, and P(A‐a)O_2_. For patients after extubation, arterial blood gas analysis was performed at the preset time points (6 h, 12 h, 18 h, and 24 h postoperatively). FiO_2_ was recorded in real time: For nasal cannula oxygen therapy, FiO_2_ was calculated as 21% + 4% × oxygen flow rate (L/min); for mask and HFNC oxygen therapy, FiO_2_ was directly read from the device setting. PaO_2_ was measured directly from arterial blood gas samples. (2) Pulmonary complications: postoperative atelectasis (diagnosed by chest X‐ray), pulmonary infections, and the number of patients requiring HFNC oxygen therapy after extubation.

The secondary outcome measures included the following: (1) Inflammatory factors: Plasma levels of TNF‐α, IL‐6, IL‐8, and IL‐10 at preanesthesia induction (T1), postinduction (T2), aortic declamping (T3), postextubation (T4), and 24 h postoperatively (T5). (2) Endothelial glycocalyx (eGC) injury markers: Plasma levels of syndecan‐1 (SDC‐1) and heparan sulfate (HS) at multiple time points. (3) Postoperative complications and rehabilitation: Number of patients requiring postoperative rescue analgesia, VAS score values, incidence of postoperative nausea and vomiting (PONV) and delirium (CAM‐ICU), extubation time, duration of ICU stay, postoperative hospital stay, and in‐hospital and 3‐month postoperative mortality.

Blood samples (5 mL) were collected into anticoagulant tubes at the following time points: before anesthesia induction (T1), after anesthesia induction and before the start of surgery (T2), during aortic declamping (T3), immediately after extubation at the end of surgery (T4), and 24 h postoperatively (T5). Plasma was immediately separated by centrifugation after blood collection and stored at −80°C until analysis. The serum levels of TNF‐α (JL10208), IL‐6 (JL14113), IL‐8 (JL19291), IL‐10 (JL19246), SDC‐1 (JL33252), and HS (JL19659) were measured using commercial ELISA kits (Jianglai Bio‐Technology, Shanghai, China).

### 2.5. Statistical Analysis

Statistical analyses were performed using SPSS software (Version 26.0). Normally distributed continuous data are presented as the mean ± standard deviation (Mean ± SD). For comparisons among multiple groups, one‐way analysis of variance (ANOVA) was used, followed by the Tukey HSD test for multiple comparison correction. A two‐way repeated‐measures ANOVA was conducted to evaluate the effects of group (between‐subjects factor) and time (within‐subjects factor) on the outcome measures. In cases of a significant interaction between time and group, simple effects analysis was performed to examine group differences at each time point, with post hoc comparisons corrected using the Bonferroni method. Skewed data are expressed as the median with interquartile range [M (Q1‐Q3)] and were analyzed using the rank‐sum test for between‐group comparisons. Categorical data were analyzed using the chi‐squared test or Fisher’s exact test, as appropriate. A *p* value < 0.05 was considered to indicate a statistically significant difference.

## 3. Results

### 3.1. General Data

**FIGURE 1:**
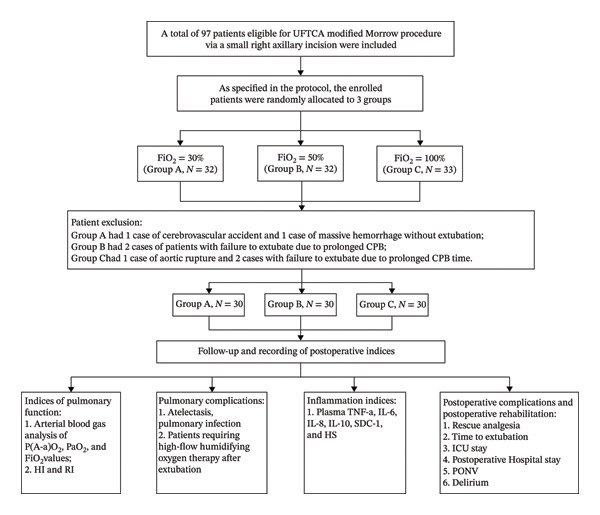
Flowchart of the study abbreviations: UFTCA, ultra‐fast‐track cardiac anesthesia; PEEP, positive end‐expiratory pressure; CPB, cardiopulmonary bypass; HI, Horowitz Index; RI, respiratory index; ICU, intensive care unit; PONV, postoperative nausea and vomiting.

This study included a total of 97 eligible patients. In Group A, one patient experienced a cerebrovascular accident during surgery and the tracheal tube could not be withdrawn in another patient due to major bleeding, resulting in both being withdrawn from the trial. In Group B, UFTCA was abandoned in two patients due to prolonged CPB time. In Group C, a patient sustained aortic rupture during aortic cannulation, while extubation failed and UFTCA was abandoned in two patients due to prolonged CPB time. Thus, a total of 90 patients were included in the final analysis, with 30 patients in each of the three groups (Figure 1). No patients were lost to follow‐up or excluded from outcome assessments at any postoperative time point. All statistical analyses were performed on the 90 patients after excluding 7 patients who withdrew from the trial, with 30 patients in each group.

There were no significant differences among the three groups in terms of age, sex, height, weight, BMI, ASA grade, NYHA grade, diastolic function grade, left ventricular outflow tract gradient (LVOTG), ejection fraction, surgical duration, CPB time, and aortic cross‐clamping time, pre‐existing disease (*p* > 0.05) (Table 1).

**TABLE 1:** General data and surgical information.

Variables	Group A (*N* = 30)	Group B (*N* = 30)	Group C (*N* = 30)	F/H/*χ* ^2^	*p* value
Age (years, Mean ± SD)	46.9 ± 13.81	46.27 ± 16.13	47.43 ± 15.68	0.04	0.95
Sex ratio (male/female) (*n*)	20/10	16/14	18/12	1.11	0.57
Height (cm, Mean ± SD)	169.10 ± 9.70	164.40 ± 8.65	165.73 ± 7.70	2.27	0.10
Weight (kg, Mean ± SD)	68.28 ± 16.08	67.34 ± 11.78	67.86 ± 12.20	0.03	0.96
BMI (kg/m^2^, Mean ± SD)	24.41 ± 3.15	24.82 ± 3.12	24.65 ± 3.57	0.11	0.89
ASA Grade III) (*n*/%)	30 (100)	30 (100)	30 (100)		
NYHA Grade (I/II/III) (*n*)	4/22/4	1/21/8	1/26/3	6.4	0.17
Diastolic function grade (II) (*n*/%)	30 (100)	30 (100)	30 (100)		
LVOTG (mmHg, Mean ± SD)	84.13 ± 19.56	79.80 ± 15.31	77.93 ± 13.83	1.12	0.32
Ejection fraction (%, Mean ± SD)	69.30 ± 4.77	69.30 ± 3.71	70.03 ± 4.10	0.28	0.75
Surgery duration (min, M [Q1–Q3])	265 (242.5–308.8)	270 (256.2–321.2)	270 (231.2–308.8)	1.20	0.54
CPB time (min, M [Q1–Q3])	115.5 (108.0–131.0)	119.5 (103.0–138.0)	111.5 (97.2–125.8)	1.68	0.43
Aortic cross‐clamping time (min, M [Q1–Q3])	77 (68.5–91.8)	71.5 (65.2–85.2)	77 (62.2–87.2)	0.90	0.63

*Pre-existing disease*					
Hypertension (*n*/%)	7 (23.3)	5 (16.7)	7 (23.3)	0.53	0.76
Diabetes (*n*/%)	2 (6.7)	1 (3.3)	2 (6.7)	0.42	0.80
Atrial fibrillation (*n*/%)	1 (3.3)	2 (6.7)	1 (3.3)	0.52	0.77
Coronary heart disease (*n*/%)	1 (3.3)	2 (6.7)	4 (13.3)	2.16	0.33
History of cerebral infarction (*n*/%)	2 (6.7)	0 (0)	0 (0)	4.0	0.12
Severe subgroup 1 of PH (*n*/%)	1 (3.3)	2 (6.7)	0 (0)	2.06	0.35

*Note:* Group A, FiO2 = 30%; Group B, FiO2 = 50%; Group C, FiO2 = 100%.

Abbreviations: ASA, American Society of Anesthesiologists; CPB, cardiopulmonary bypass; BMI, body mass index; LVOTG, left ventricular outflow tract gradient; NYHA, New York Heart Association; PH, pulmonary hypertension.

### 3.2. Intraoperative Opioid Use and Analgesic Effect

There were no differences in the total sufentanil and remifentanil doses used intraoperatively, the number of patients requiring postoperative rescue analgesia, tramadol dosage, and the postoperative VAS scores among the three groups (*p* > 0.05) (Table 2).

**TABLE 2:** Intraoperative opioid use and postoperative analgesia.

Variables	Group A (*N* = 30)	Group B (*N* = 30)	Group C (*N* = 30)	F/H/*χ* ^2^	*p* value
Intraoperative sufentanil (ug, M [Q1–Q3])	60 (50–70)	57.5 (47.5–67.5)	60 (49–71)	3.93	0.14
Intraoperative remifentanil (mg, M [Q1–Q3])	2.6 (1.8–3.5)	2.9 (1.9–3.8)	2.5 (1.7–3.4)	4.36	0.11
Rescue analgesia in ward (*n*/%)	16 (53.3)	12 (40.0)	15 (50.0)	1.15	0.56
Rescue analgesia in ICU (*n*/%)	1 (3.3)	0 (0)	0 (0)	2.02	0.36
Tramadol dosage (mg, M (Q1–Q3))	100 (25–150)	50 (0–200)	75 (0–100)	1.48	0.48
VAS score (points, M [Q1–Q3])	3 (2.0–4.0)	2 (1.0–3.0)	3 (2.0–4.0)	4.93	0.08

*Note:* Group A, FiO_2_ = 30%; Group B, FiO_2_ = 50%; Group C, FiO_2_ = 100%.

Abbreviations: ICU, intensive care unit; VAS, visual analog scale.

### 3.3. Primary Outcomes: Pulmonary Function and Postoperative Pulmonary Complications

In Group A, the HI was higher than in Group C at D3 and D6 (*p* <0.05). There were no statistically significant differences in HI at D3 and D6 between Groups B and C (*p* > 0.05). The P(A‐a)O_2_ in Group A was lower than that in Group C at D5 and D6 (*p* < 0.05), while there were no significant differences between Groups B and C (*p* > 0.05). The RI in Group A was lower than that in Group C at D6 (*p* < 0.05), with no significant differences between Groups B and C (*p* > 0.05). There were no differences in HI, P(A‐a)O_2_, and RI between Groups A and B at all time points (*p* > 0.05) (Table 3).

**TABLE 3:** Indices of pulmonary function at different postoperative time points.

Variables	Group	D1 (*n* = 30)	D2 (*n* = 30)	D3 (*n* = 30)	D4 (*n* = 30)	D5 (*n* = 30)	D6 (*n* = 30)
HI (mmHg)	A	409.37 ± 51.76	259.63 ± 121.00	328.90 ± 117.06^∗^	352.67 ± 112.35	355.71 ± 107.78	357.70 ± 95.70^∗^
	B	382 ± 52.43	248.81 ± 111.57	314.17 ± 78.23	314.67 ± 109.09	316.97 ± 104.12	331.60 ± 100.85
	C	397.94 ± 42.14	215.79 ± 97.51	260.80 ± 115.00	288.37 ± 108.53	293.51 ± 86.28	294.63 ± 76.47

P(A‐a)O_2_ (mmHg)	A	24.52 ± 9.03	166.93 ± 56.39	129.14 ± 63.21	110.01 ± 70.46	88.39 ± 65.87^∗^	81.89 ± 66.62^∗^
	B	21.45 ± 10.05	171.76 ± 59.76	122.12 ± 60.03	124.10 ± 69.91	107.93 ± 66.87	94.42 ± 52.97
	C	18.05 ± 8.26	165.17 ± 58.16	163.02 ± 58.73	145.27 ± 83.82	134.59 ± 57.24	121.28 ± 53.52

RI	A	0.29 ± 0.35	1.68 ± 1.25	1.26 ± 1.37	0.98 ± 0.86	0.85 ± 0.78	0.72 ± 0.64^∗^
	B	0.27 ± 0.15	2.02 ± 1.95	1.05 ± 0.68	1.18 ± 0.89	1.06 ± 0.81	0.92 ± 0.68
	C	0.22 ± 0.12	2.23 ± 1.71	1.52 ± 1.08	1.33 ± 0.88	1.22 ± 0.67	1.15 ± 0.64

*Note:* Group A, FiO_2_ = 30%; Group B, FiO_2_ = 50%; Group C, FiO_2_ = 100%; D1, preoperatively; D2, postoperatively on ICU admission; D3, 6 h postoperatively; D4, 12 h postoperatively; D5, 18 h postoperatively; D6, 24 h postoperatively.

Abbreviations: HI, Horowitz Index; RI, respiratory index.

^∗^ *p* < 0.05 vs. Group C.

No statistically significant differences were observed in postoperative atelectasis, pulmonary infections, or high‐flow oxygen therapy rates in the ICU after extubation among the three groups (*p* > 0.05) (Table 4).

**TABLE 4:** Postoperative pulmonary complications.

Variables	Group A (*N* = 30)	Group B (*N* = 30)	Group C (*N* = 30)	*χ* ^2^	*p* value
Atelectasis (*n*/%)	5 (16.7)	8 (26.7)	5 (16.7)	1.25	0.53
Pulmonary infection (*n*/%)	5 (16.7)	6 (20.0)	7 (23.3)	0.41	0.81
HFNC in the ICU (*n*/%)	4 (13.3)	7 (23.3)	9 (30.0)	2.44	0.29

*Note:* Group A, FiO2 = 30%; Group B, FiO2 = 50%; Group C, FiO2 = 100%.

Abbreviations: ICU, intensive care unit; HFNC, High‐flow nasal cannula.

### 3.4. Secondary Outcomes: Inflammatory Factors and eGC Injury Markers

Group A had lower TNF‐α levels at T3 to T5 (*p* < 0.05), and Group B showed lower TNF‐α levels at T5 (*p* < 0.05) compared to Group C. Group A had lower TNF‐α levels than Group B at T3 and T4 (*p* < 0.05) (Figure 2(a), Table 5). Groups A and B exhibited lower IL‐6 levels at T5 compared to Group C (*p* < 0.05), while no significant difference was observed in the IL‐6 concentration between Groups A and B at T5 (*p* > 0.05) (Figure 2(b), Table 5). Group A showed lower HS levels at T5 (*p* < 0.05) than Group C, while no significant difference was found in HS levels between Groups A and B at T5 (*p* > 0.05) (Figure 2(c), Table 5). The SDC‐1 concentration was lower in Groups A and B than in Group C at T5 (*p* < 0.05), and no significant difference was found in SDC‐1 concentration between Groups A and B at T5 (*p* > 0.05) (Figure 2(d), Table 5). There were no significant differences in IL‐8 and IL‐10 levels at T1 to T5 among the three groups (*p* > 0.05) (Table 5).

FIGURE 2Plasma inflammatory factors and eGC injury markers. (a) Plasma TNF‐α. (b) Plasma IL‐6. (c) Plasma HS. (d) Plasma SDC‐1. Note: ^∗^ *p* < 0.05 vs. Group C; ^#^ *p* < 0.05 vs. Group B; Note: Group A, FiO_2_ = 30%; Group B, FiO_2_ = 50%; Group C, FiO_2_ = 100%; T1, preanesthesia induction; T2, postinduction, T3, aortic declamping; T4, postextubation; T5, 24 h postoperatively.

**Figure:**
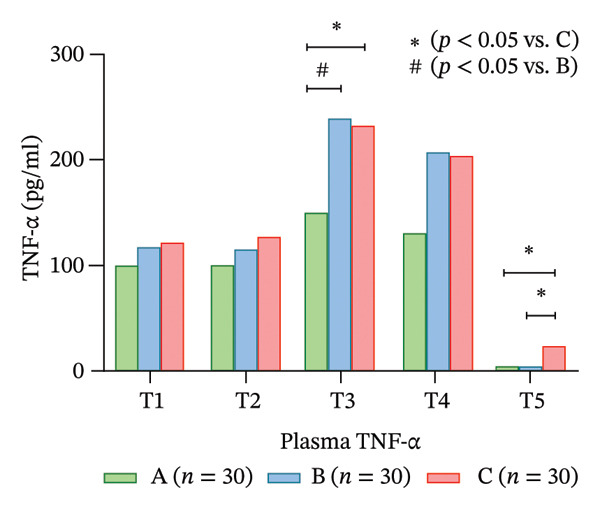
(a)

**Figure:**
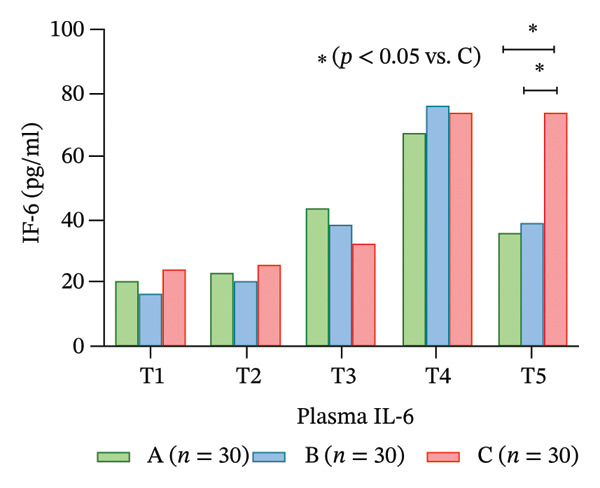
(b)

**Figure:**
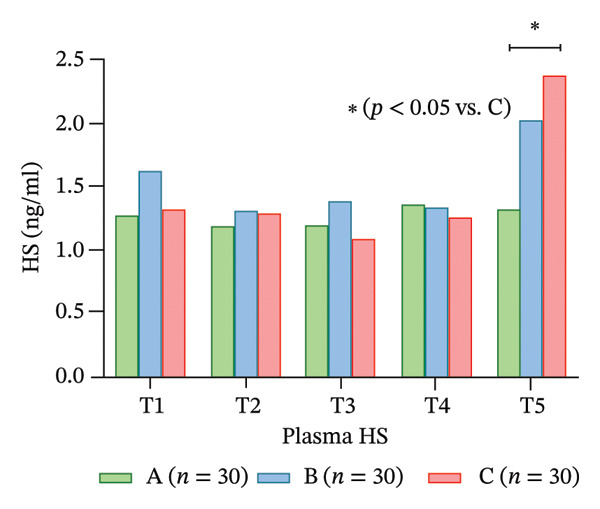
(c)

**Figure:**
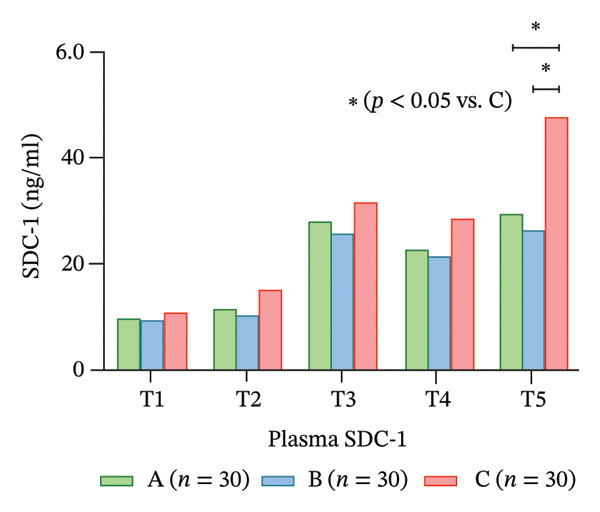
(d)

**TABLE 5:** Plasma inflammatory factors and eGC injury.

Variables	Group	T1 (*n* = 30)	T2 (*n* = 30)	T3 (*n* = 30)	T4 (*n* = 30)	T5 (*n* = 30)
TNF‐a (pg/mL, Mean ± SD)	A	101.31 ± 55.77	101.48 ± 45.32	152.55 ± 76.99^∗#^	132.77 ± 76.25	6.23 ± 4.54^∗^
	B	118.41 ± 24.31	115.71 ± 11.75	243.21 ± 61.71	210.41 ± 92.89	5.95 ± 4.54^∗^
	C	122.41 ± 41.05	129.5 ± 52.36	234.24 ± 99.45	207.91 ± 90.75	26.07 ± 7.94

IL‐6 (pg/mL, Mean ± SD)	A	20.81 ± 3.59	23.41 ± 8.39	44.21 ± 30.09	68.5 ± 33.66	37.27 ± 9.4^∗^
	B	16.89 ± 9.48	21.15 ± 37.44	39.57 ± 38.56	77.12 ± 46.9	40.65 ± 18.88
	C	24.19 ± 12.7	25.81 ± 14.37	32.43 ± 26.04	75.22 ± 53.1	74.76 ± 57.42

IL‐8 (pg/mL, Mean ± SD)	A	6.55 ± 2.5	6.21 ± 1.86	23.81 ± 4.57	27.67 ± 9.56	15.54 ± 3.73
	B	7.04 ± 8.99	5.2 ± 4.09	21.39 ± 14.18	23.5 ± 14.3	13.86 ± 6.16
	C	4.78 ± 2.31	4.44 ± 2.31	16.49 ± 16.05	23.52 ± 17.27	14.53 ± 12.02

IL‐10 (pg/mL, Mean ± SD)	A	7.55 ± 2.27	7.99 ± 2.86	17.3 ± 8.86	19.19 ± 5.66	11.84 ± 5.99
	B	7.23 ± 3.72	6.58 ± 3.22	13.97 ± 6.7	16.04 ± 6.97	11.63 ± 4.69
	C	6.49 ± 2.85	7.39 ± 3.17	16.1 ± 13.39	21.26 ± 15.38	12.58 ± 11.43

HS (pg/mL, Mean ± SD)	A	1.3 ± 0.39	1.23 ± 0.39	1.24 ± 0.33	1.38 ± 0.66	1.36 ± 0.52^∗^
	B	1.65 ± 0.62	1.32 ± 0.39	1.43 ± 0.36	1.37 ± 0.41	2.03 ± 1.03
	C	1.37 ± 0.71	1.31 ± 0.6	1.11 ± 0.51	1.27 ± 0.69	2.38 ± 0.84

SDC‐1 (pg/mL, Mean ± SD)	A	10.3 ± 2.4	11.98 ± 4.42	28.67 ± 8.56	22.83 ± 7	30.05 ± 9.34^∗^
	B	10.86 ± 5.12	10.85 ± 5.69	26.38 ± 14.37	21.99 ± 10.78	26.78 ± 13.32^∗^
	C	11.54 ± 5.16	15.7 ± 8.94	32.28 ± 18.33	29.32 ± 14.32	47.9 ± 15.77

*Note:* Group A, FiO_2_ = 30%; Group B, FiO_2_ = 50%; Group C, FiO_2_ = 100%; T1, preanesthesia induction; T2, postinduction, T3, aortic declamping; T4, postextubation; T5, 24 h postoperatively.

^∗^ *p* < 0.05 vs. Group C.

^#^ *p* < 0.05 vs. Group B.

### 3.5. Secondary Outcomes: Comparison of Postoperative Rehabilitation and Other Postoperative Complications

No significant differences were observed among the three groups in terms of the awakening time after surgery, time to tracheal extubation, duration of ICU stay, and length of hospital stay (*p* > 0.05). The incidence rates of PONV and delirium were not statistically different among the three groups (*p* > 0.05). There were no instances of secondary surgery, reintubation, in‐hospital deaths, or deaths within 3 months after surgery in any of the three groups (Table 6).

**TABLE 6:** Postoperative rehabilitation and other postoperative complications.

Variables	Group A (*N* = 30)	Group B (*N* = 30)	Group C (*N* = 30)	H/*χ* ^2^	*p* value
Time to emergence (min, M [Q1–Q3])	5 (3.0–8.0)	4 (2.0–11.0)	3.5 (2.0–7.5)	0.93	0.62
Time to extubation (min, M [Q1–Q3])	7 (4.0–15.0)	10 (5.0–22.0)	5.5 (3.0–11.5)	2.00	0.36
ICU stay (*h*, M [Q1–Q3])	22.0 (18.0–42.1)	24.5 (19.5–48.4)	21.5 (17.2–49.2)	0.29	0.86
Hospital stay (*d*, M [Q1–Q3])	8.0 (7.0–10.0)	9.0 (7.0–12.0)	8.5 (7.0–11.5)	5.17	0.07
PONV (*n*/%)	6 (20.0)	6 (20.0)	10 (33.3)	1.92	0.38
Delirium (*n*/%)	0 (0)	0 (0)	1 (3.3)	2.02	0.36
Reoperation (*n*/%)	0 (0)	0 (0)	0 (0)		
Reintubation (*n*/%)	0 (0)	0 (0)	0 (0)		
In‐hospital mortality (*n*/%)	0 (0)	0 (0)	0 (0)		
Postoperative 3‐month mortality rate (*n*/%)	0 (0)	0 (0)	0 (0)		

*Note:* Group A, FiO_2_ = 30%; Group B, FiO_2_ = 50%; Group B, FiO2 = 100%.

Abbreviation: ICU, intensive care unit; PONV, postoperative nausea and vomiting.

## 4. Discussion

In this study, we primarily compared the effects of different FiO_2_ (30%, 50%, and 100%) administered during CPB on postoperative pulmonary function and pulmonary complications in patients undergoing the modified Morrow procedure via a right axillary mini‐incision under a standardized ultra‐low VT lung‐protective strategy. The use of a protective ventilation strategy combining 30% FiO_2_ with minimal VT, compared with the 100% FiO_2_ group, promoted early postoperative oxygenation improvement. The underlying mechanism may be associated with the early resolution of inflammation, and this strategy did not increase postoperative pulmonary complications.

Pulmonary function in patients undergoing cardiac surgery with CPB is influenced by multiple factors [9], including advanced age, cardiac function status, underlying pulmonary disease, CPB duration, operative time, surgical outcomes, anesthesia management, and postoperative pain control. There were no statistically significant differences in these parameters among the three groups in this study, thereby minimizing their potential confounding effects on the study results to a certain extent.

There is currently an ongoing debate regarding the optimal intraoperative FiO_2_. The 2020 Clinical Expert Consensus on Perioperative Protective Ventilation Strategies [10] recommends that, while maintaining adequate oxygenation, the use of pure oxygen and unnecessarily high FiO_2_ should be avoided during mechanical ventilation. It suggests adjusting FiO_2_ to < 40% and reducing it to the lowest possible level. In this study, the HI at 6 and 24 h postoperatively was significantly higher in the 30% FiO_2_ group than in the 100% FiO_2_ group. The P(A‐a)O_2_ at 18 and 24 h postoperatively was significantly lower in the 30% FiO_2_ group than in the 100% FiO_2_ group. Consistently, the RI at 24 h postoperatively was significantly lower in the 30% FiO_2_ group than in the 100% FiO_2_ group. These findings suggest that the ultra‐low VT lung‐protective strategy combining 30% FiO_2_ exerts a positive effect on early postoperative pulmonary function recovery in patients undergoing modified Morrow procedure with UFTCA. The observed results may be attributed to the following mechanisms. High‐concentration oxygen (100%) is rapidly taken up by hemoglobin within the alveoli, predisposing to absorption atelectasis [11]. Hyperoxia also induces excessive generation of reactive oxygen species, activating inflammatory pathways and promoting the release of proinflammatory cytokines such as TNF‐α, IL‐6, and IL‐8. The ensuing inflammatory response damages the alveolar–capillary barrier, increases pulmonary vascular permeability, and leads to interstitial edema and impaired gas exchange. In contrast, low oxygen concentration (30%) may promote early resolution of inflammation by preserving the activity of endogenous antioxidant systems. Additionally, hypoxia may attenuate ischemia–reperfusion injury induced by CPB [12].

Previous studies have shown that, compared with the nonventilation group during CPB, the ventilation group during CPB can reduce immune‐inflammatory responses and help improve postoperative oxygenation [13–15]. In this study, serum TNF‐α and IL‐6 levels in all three groups showed an initial increase followed by a subsequent decrease, which is consistent with the findings of Yuan and Allen [16, 17]. Compared with the 100% FiO_2_ group, the 30% FiO_2_ group exhibited significantly lower plasma TNF‐α and IL‐6 levels at 24 h postoperatively. The changes in HS and SDC‐1 were consistent with the above results. These results indicate that the use of a low inspired oxygen concentration during CPB can attenuate the inflammatory response and reduce the increase in pulmonary vascular permeability, thereby exerting a lung‐protective effect. These findings are also consistent with the aforementioned observation that 30% FiO_2_ promotes early recovery of pulmonary oxygenation.

Consistent with the findings of Salter [18], in this study, there were no significant differences in postoperative pulmonary complications (including atelectasis, pneumonia, and high‐flow oxygen therapy in the ICU) among the three groups. This may be attributed to the fact that all three groups received the same ultra‐low VT, low‐frequency ventilation, and PEEP strategy, which itself has been shown to significantly reduce the incidence of postoperative pulmonary complications. Given that protective ventilation already exerted a predominant protective effect, the difference in FiO_2_ alone may not have been sufficient to yield additional statistical significance in complication rates. Additionally, the overall incidence of postoperative pulmonary complications in this study was low, and the sample size may have lacked sufficient power to detect statistical significance. There were also no statistically significant differences among the three groups in terms of secondary outcomes, such as postoperative rehabilitation and other postoperative complications. This result may also be related to the sample size, as discussed above.

The present study has several limitations. The single‐center design and small sample size may introduce selection bias and limit statistical power. Ultra‐low VT ventilation (1.5 mL/kg) may fall below dead space ventilation, and 100% FiO_2_ may cause absorption atelectasis, potentially reducing the benefits of CPB ventilation and affecting between‐group comparisons. A blank control group with ventilation suspension was not designed, and no preoperative lung function tests were collected. Finally, the exclusion criteria were predefined according to our UFTCA protocol, targeting patients eligible for early extubation to ensure a comparable spontaneous breathing state for outcome assessment. Consequently, patients at higher risk for postoperative pulmonary complications were excluded by design, potentially biasing the results and limiting the generalizability of our findings to higher risk populations.

## 5. Conclusion

Compared with 100% FiO_2_, 30% FiO_2_ combined with ultra‐low VT ventilation did not increase the incidence of postoperative complications in this cohort. Moreover, patients in the 30% FiO_2_ group had higher HI and lower P(A‐a)O_2_ and RI postoperatively. These findings indicate that, in patients undergoing modified Morrow procedure via a small right axillary incision, the use of 30% FiO_2_ with ultra‐low VT ventilation during CPB was associated with better early postoperative oxygenation and lower inflammatory marker levels, without an observed increase in postoperative pulmonary complications. Further validation in larger studies is warranted.

## Author Contributions

Linting Xu and Lixin Wang contributed to the conception and design of the study; Linting Xu and Lixin Wang contributed to the interpretation and analysis of the data; Linting Xu, Tian Jiang, and Lixin Wang contributed to data collection and drafting the manuscript; Meijuan Yan, Hanwei Wei, Haozhou Wang, Jinchen Guo, and Yong Cui contributed to the conduct of the study.

## Funding

The study was supported by the National Science and Technology Major Project for Noncommunicable Chronic Diseases (No. 2024ZD0527205‐3), the Health Science Technology Plan Project of Zhejiang Province (Nos. 2022KY037, 2022KY062, 2024KY009, 2024KY657, and 2024KY779), and the Seed Fund Project of Zhejiang Provincial People’s Hospital (No. ZRY2022J011).

## Conflicts of Interest

The authors declare no conflicts of interest.

## Data Availability

The datasets are available from the corresponding author upon reasonable request.
